# Innovative Therapeutic Endoscopy of the Upper Gastrointestinal Tract: Review of the Japan Gastroenterological Endoscopy Society Core Sessions

**DOI:** 10.1002/deo2.70385

**Published:** 2026-07-28

**Authors:** Mikitaka Iguchi, Tomoki Michida, Yasuhiko Abe, Hisashi Doyama, Noriya Uedo, Hajime Isomoto, Masayuki Ohta, Junko Fujisaki

**Affiliations:** ^1^ Second Department of Internal Medicine Wakayama Medical University Wakayama Japan; ^2^ Department of Gastrointestinal Oncology Osaka International Cancer Institute Osaka Japan; ^3^ Division of Endoscopy Yamagata University Hospital Yamagata Japan; ^4^ Department of Gastroenterology Ishikawa Prefectural Central Hospital Kanazawa Japan; ^5^ Division of Gastroenterology and Nephrology Tottori University Faculty of Medicine Tottori Japan; ^6^ Research Center for GLOBAL and LOCAL Infectious Diseases Oita University Oita Japan; ^7^ Cancer Institute Hospital of the Japanese Foundation for Cancer Research Tokyo Japan

**Keywords:** collaborative treatment with surgery, endoscopic resection, gastrointestinal strictures, therapeutic endoscopy, upper gastrointestinal bleeding

## Abstract

The Japanese Gastroenterological Endoscopy Society (JGES) established four core sessions on endoscopic treatment for upper gastrointestinal diseases under the theme ‘Innovative Therapeutic Endoscopy’ between 2023 and 2024. This review summarizes these sessions and compiles them as a conference report. At the 105th JGES Core Session, 14 studies were presented as frontiers of treatment for upper gastrointestinal stenosis. At the 106th JGES Core Session, 13 research findings were presented as developments of new endoscopic techniques for the management of neoplastic lesions in the upper digestive tract. At the 107th JGES Core Session, reports were presented from 14 institutions concerning innovations in therapeutic endoscopy, including endoscopic treatment advancements for upper gastrointestinal diseases in internal medicine and surgery. At the 108th JGES Core Session, research from 11 institutions was presented on endoscopic treatment for upper gastrointestinal bleeding. These four core sessions enabled us to recognize the current state of treatment and management approaches for neoplastic lesions, strictures, and bleeding in the upper gastrointestinal tract, and to clarify future challenges. We hope this will contribute to the advancement of upper gastrointestinal endoscopic therapy.

## 1 Introduction

The Japanese Gastroenterological Endoscopy Society (JGES) held four symposia addressing various challenges faced in endoscopic treatment of upper gastrointestinal diseases. The chairs, topics, and number of accepted presentations for each session are shown in Table 1. They included:

- 105th core session: Innovative therapeutic endoscopy: Frontiers of treatment for upper gastrointestinal stenosis. Chairmen: Hajime Isomoto, Yasuhiko Abe;
- 106th core session: Development of new endoscopic techniques for the management of neoplastic lesions in the upper digestive tract. Chairmen: Junko Fujisaki, Noriya Uedo;
- 107th core session: Innovation therapeutic endoscopy: Endoscopic treatment advancements for upper gastrointestinal diseases in internal medicine and surgery. Chairmen: Masayuki Ohta, Hisashi Doyama;
- 108th core session: Endoscopic treatment of upper gastrointestinal bleeding. Chairmen: Tomoki Michida, Mikitaka Iguchi.

**TABLE 1:** Chairpersons and topics of each Japanese Gastroenterological Endoscopy Society (JGES) Congress.

Congress	Chairmen	Topics	Number of research presentations
**105th**	Hajime Isomoto (Division of Gastroenterology and Nephrology, Tottori University Faculty of Medicine Yasuhiko Abe (Division of Endoscopy, Yamagata University Hospital)	Innovative therapeutic endoscopy: Frontiers of treatment for upper gastrointestinal stenosis	14
**106th**	Junko Fujisaki (Cancer Institute Hospital of Japanese Foundation for Cancer Research) Noriya Uedo (Osaka International Cancer Institute)	Development of new endoscopic techniques for the management of neoplastic lesions in the upper digestive tract	13
**107th**	Masayuki Ohta (Research Center for GLOBAL and LOCAL Infectious Diseases, Oita University) Hisashi Doyama (Department of Gastroenterology, Ishikawa Prefectural Central Hospital)	Innovation therapeutic endoscopy: Endoscopic treatment advancements for upper gastrointestinal diseases in internal medicine and surgery	14
**108th**	Tomoki Michida (Osaka International Cancer Institute) Mikitaka Iguchi (Second Department of Internal Medicine, Wakayama Medical University)	Endoscopic treatment of upper gastrointestinal bleeding	11

Abstracts were solicited focusing on gastrointestinal strictures, the newest treatments for neoplastic lesions, collaborative treatment with surgery, and gastrointestinal bleeding. Research selected from numerous submissions was rigorously evaluated and accepted.

The contents of each session are outlined in the following sections.

### 1.1 Frontiers of Treatment for Upper Gastrointestinal Stenosis (The 105th JGES Core Session)

A total of 14 presentations were accepted from 23 abstract submissions in this symposium. Nine of them were reports on post‐endoscopic submucosal dissection (post‐ESD) strictures in superficial esophageal squamous cell carcinoma (ESCC). Prior to each presentation, Dr. Naoyuki Yamaguchi gave a keynote lecture on the current status and challenges of endoscopic treatment for upper GI strictures, with recent advances.

Risk factors for post‐esophageal ESD stricture were investigated by Mizuno et al. and Ueda et al. They identified several risk factors, including a history of chemoradiotherapy (CRT), whole circumferential resection, and lesions at the cervical/upper esophagus. Notably, the width of the residual esophageal mucosa was a better predictor of post‐ESD stricture than the circumference of mucosal defects.

Habu et al. conducted a multicenter, on‐line survey to evaluate the treatment strategy for esophageal ESD with mucosal defects greater than 1/2 and less than 3/4 of the circumference, for which a prophylactic treatment to avoid post‐ESD stricture has not been actively recommended in the current clinical guideline [1]. Several institutions answered that an esophageal stricture occurred in more than 10%–30% of cases, and that prophylactic treatment, mainly using local steroid injection, was provided to more than 50% of patients. It was also found that dysphagia developed, and there was interference with ESD for metachronous regions.

Three reports focused on conventional local steroid injection or oral steroid intake to prevent post‐esophageal ESD stricture. Abe et al. summarized the results of a phase III study of oral steroid intake versus local steroid injection (JCOG1217) [2]. Superficial ESCCs greater than 1/2 of the circumference, non‐whole circumference, and less than 50 mm in longitudinal diameter were included. There was no statistically significant difference in the stricture‐free survival rate 12 weeks after ESD between the two groups (local steroid 88.5% vs. oral steroid 94.8%). Based on no significant superiority of oral steroids to local steroids, they concluded that local steroids were recommended as a standard prophylactic treatment. In a report by Ono et al. investigating the add‐on effect of oral steroid intake to local steroid injection, there was no difference in stricture occurrence with or without additional oral steroid. However, the combined use of both reduced the number of endoscopic balloon dilatations (EBDs) required to release the stricture, especially in patients with mucosal defects greater than 9/10 of the circumference. Taniguchi et al. reported short‐ and long‐term outcomes of combination therapy with prophylactic EBD and oral steroids. The success rate for releasing post‐ESD strictures was 74.0%, with no adverse events. The number of EBDs required after the procedure decreased significantly in the stricture‐release success group. The 3‐year overall survival rate also tended to be longer in the stricture‐release success group than in the stricture‐release no‐success group, potentially contributing to an improved prognosis.

Several novel and unique methods for preventing post‐esophageal ESD stricture were reported by Goto et al., Shibagaki et al., and Sakaguchi et al. Local steroid injection using a spray tube would be promising as a safer and easier method than the conventional method using a local injection needle, with a lower risk of muscularis propria damage. In a report using triamcinolone acetonide (TA) filling methods, there was no difference in the occurrence of post‐ESD stricture and the number of EBDs required between mucosal defects involving the whole circumference of the esophagus, ≤49 mm, and ≥50 mm in longitudinal diameter [3]. This suggested the possibility of modifying the current recommendation of endoscopic resection for ESCC [1]. The combination therapy of local steroid injection and mucosal defect shielding using polyglycolic acid (PGA) sheets significantly reduced the risk of developing post ESD stricture by appropriately 60% compared with no treatment, which was superior to local steroid injection alone and PGA sheet shielding alone [4]. Periodic local steroid injection was also found to be effective for preventing EBD‐refractory strictures.

There were also several reports on benign esophageal strictures, post‐duodenal ESD strictures, and malignant esophageal and gastroduodenal strictures. Toshimori et al. reported a novel endoscopy‐assisted balloon dilatation method, named “side‐by‐side EBD,” to improve symptom relapse in patients with esophageal achalasia treated by per oral endoscopic myotomy or surgical myotomy [5]. This procedure can safely and gradually dilate the esophagogastric junction to a diameter of 20–30 mm or more under direct endoscopic visualization in a retroflexed position by delivering and inflating the conventional through‐the‐scope balloon along the outside of the endoscope. In pilot data obtained from eight procedures in six patients, good treatment outcomes were seen with no severe adverse events. Mitani et al. investigated the usefulness of radical incision and cutting (RIC) for refractory benign esophageal stricture [6]. There was no significant difference in the improvement of the dysphagia score (84.4% vs. 89.5%), cumulative patency rate (58.5% vs. 41.1%, after 12 months), and the frequency of repeated RIC (40.6% vs. 42.1%) between postoperative anastomotic strictures and nonoperative strictures, but the cumulative complete stricture‐free rate was significantly lower for nonoperative strictures (75.4% vs. 52.2%, after 24 months). Esophageal perforation occurred in 6.3% of the postoperative stricture group. They concluded that RIC could be a promising and safe treatment even for non‐operative esophageal strictures. Kubosawa et al. retrospectively validated their treatment strategy for post‐ESD mucosal defects with superficial non‐ampullary duodenal epithelial tumors, in which the mucosal defect was longitudinally sutured in lesions beyond the superior duodenal angle (group D) and not in lesions with the bulb (group B) [7]. In group D, post‐ESD stricture occurred in 1% of patients without suturing, but not in patients with suturing. In group B, post‐ESD stricture occurred in 15% of the patients, all of whom had large mucosal defects involving the pyloric ring.

A systematic meta‐analysis by Hamada et al. showed that esophageal stenting might be inferior to gastrostomy in overall survival, with an increased risk of severe adverse events, especially in patients who received radiation therapy. Esophageal stenting should be performed carefully in patients who strongly prefer oral intake after fully informed consent is obtained. A multicenter, prospective study by Toki et al. evaluated the efficacy and safety of a highly flexible metallic stent (WallFlex Duodenal Soft) for malignant gastric outlet obstruction [8]. A total of 60 patients, including those with pancreatic cancer, gastric cancer, and bile duct cancer, were enrolled. The median dilation rate increased gradually from a median of 55% on the day after the procedure to 75% at 1 week after, with a significant improvement in the dysphagia score. Adverse events such as cholecystitis/cholangitis/pancreatitis, bleeding, or perforation occurred in 25%, but no procedure‐related deaths were observed during the follow‐up period.

Finally, Dr. Takahisa Furuta summarized the reports presented in this symposium in special remarks.

### 1.2 Development of New Endoscopic Techniques for the Management of Neoplastic Lesions in the Upper Digestive Tract (the 106th JGES Core Session)

The second session of the fifth round JGES Core Session was entitled “Development of new endoscopic techniques for the management of neoplastic lesions in the upper digestive tract,” and it was held on October second during JDDW2023 in Kobe. The session was chaired by Junko Fujisaki (Cancer Institute Hospital of the Japanese Foundation for Cancer Research) and Noriya Uedo (Osaka International Cancer Institute). As background, although the usefulness of endoscopic treatment as a minimally invasive treatment for neoplastic lesions in the pharynx, esophagus, stomach, and duodenum is well established, there is still much room for improvement of the technique, including the development of treatment methods for new diseases and indications, simplification of the procedure for the dissemination of treatment methods, innovations for difficult‐to‐treat cases, and prevention and countermeasures against complications. Therefore, the aim of this session was to explore the future direction of endoscopic treatment of neoplastic lesions in the upper gastrointestinal tract by presenting as many new innovations and developments in endoscopic treatment as possible on video.

A total of 51 abstracts were submitted, and the following 13 abstracts were carefully selected: risk factors for synchronous and metachronous multiple cancers in Barrett's esophageal adenocarcinoma and feasibility of step‐wise ESD [9]; the potential efficacy of highly adhesive gelatin hydrophobic microparticles for the prevention of upper gastrointestinal ESD [10]; new innovations and developments in endoscopic local resection for gastric submucosal tumors [11]; a multicenter, randomized, controlled trial of spray‐ESD versus conventional ESD for gastric epithelial tumor (Spray‐G Trial) [12]; usefulness of ultra‐thin endoscopes for difficult esophageal ESD [13]; usefulness of extra‐lesional clip‐line traction in gastric ESD for lesions in the greater curvature of the upper stomach [14]; implementation and improvement of ESD for superficial head and neck cancer; clinical trial of the Cryoballoon Ablation System for superficial esophageal squamous cell carcinoma on scar after endoscopic resection (CRYO‐SCAR study, EPOC 1902) [15]; closure of the mucosal defect after gastric ESD using a reopenable clip over line method: a prospective feasibility study [16]; multicenter, retrospective study on different endoscopic treatment methods for duodenal neuroendocrine tumors—utility of endoscopic resection with the over‐the‐scope clip [17]; PGA sheets plus basic fibroblast growth factor (bFGF) for prevention of post‐ESD stenosis in a porcine model [18]; a study on the learning curve for a novel through‐the‐scope wound closure system [19]; and a strategy for endoscopic treatment of upper gastrointestinal submucosal tumors [20].

In particular, two multicenter, prospective studies were excellent in terms of their high level of evidence. One observational study by Nonaka et al. demonstrated the usefulness of the Cryoballoon Ablation System, which has been reported to be useful in the treatment of Barrett's esophageal lesions and in the treatment of esophageal squamous cell carcinoma on previous endoscopic treatment scars. Another multicenter, randomized, controlled trial by Maehara et al. demonstrated that gastric ESD using the spray coagulation mode significantly reduced the number of forceps coagulations for intraprocedural hemorrhage compared with conventional ESD (Spray‐G Trial). In addition, two animal studies showed outstanding novelty. Nishimura et al. showed the efficacy of combination therapy using PGA sheets and bFGF in preventing strictures after circumferential esophageal ESD in a porcine model. The study by Sasaki et al. showed the efficacy of highly adhesive gelatin hydrophobized gelatin (microparticles: hMPs) for the prevention of post‐esophageal ESD strictures and the coating closure of micro‐perforations during duodenal ESD in a mini pig model.

We expect that further evaluation of new endoscopic techniques for the management of neoplastic lesions in the upper digestive tract will be performed in well‐designed clinical studies.

### 1.3 Endoscopic Treatment Advancements for Upper Gastrointestinal Diseases in Internal Medicine and Surgery (the 107th JGES Core Session)

The core session of the 107th Japan Gastroenterological Endoscopic Society featured 14 studies focusing on the development and evaluation of endoscopic treatments for upper gastrointestinal diseases, facilitated through collaboration between internal medicine and surgery. The presentations spanned the entire upper gastrointestinal tract, with six studies focusing on the stomach, five on the duodenum, four on the esophagus, and one on the pharynx.

In the keynote lecture, Nomura et al. discussed the history and current status of laparoscopy and endoscopy cooperative surgery (LECS).

In the gastric presentations, Ohki et al. presented a combination of ESD and laparoscopic lymph node dissection (LLD) for 25 early gastric cancers. No long‐term recurrence was observed, regardless of the presence of undifferentiated adenocarcinoma, submucosal invasion, lymphovascular involvement, or lymph node metastasis. The combination of ESD and LLD appears to be an effective and minimally invasive approach that maintains long‐term quality of life for selected patients at risk for lymph node metastasis.

Both Hirasawa et al. and Uchita et al. presented findings on endoscopic resection with one‐port placement (EROPP) for a total of 46 gastric subepithelial tumors (SETs). The camera port was inserted through the umbilicus to maintain intra‐abdominal pressure. All defect holes after resection of the SETs were sutured using an over‐the‐scope clip by the endoscopists. EROPP for gastric SETs is feasible and suitable for introducing pure endoscopic full‐thickness resection (EFTR) [21]. Sato et al. reported the safety and effectiveness of LECS. None of the patients developed postoperative complications or recurrence in 14 gastric SETs. Reduced port surgery (the single port technique) was performed safely and was considered excellent in terms of cosmetic outcomes.

In the duodenum, nearly all presentations by Okugawa, Hashigami, Yamamoto, Aoyagi, and Higuchi et al. focused on LECS for superficial non‐ampullary duodenal epithelial tumors (SNADETs). The study of a total of 90 SNADETS reported an impressive en bloc resection rate of 98.8%, underscoring the effectiveness of LECS in achieving complete tumor removal. Although intraoperative adverse events occurred in 11.1% of cases and postoperative adverse events in 15.6%, they were manageable, with the most common complications being intraoperative perforation and postoperative bleeding. Only 2.2% of cases required conversion to laparotomy, demonstrating the procedure's overall safety and minimal invasiveness. Duodenal‐LECS (D‐LECS) can therefore be performed with relatively low risk due to the refinement of the D‐LECS technique [22]. Yamazaki et al. similarly reported positive outcomes with laparoscopy‐assisted, endoscopic, full‐thickness resection using a ligation device for nine duodenal neuroendocrine tumors and seven SNADETs, achieving complete resections without any adverse events. The potential to expand the indications for this technique in the future is promising. In addition, Higuchi et al. presented data showing high resection rates of LECS for 76 SETs, eight epithelial tumors, and one esophageal SET, further reinforcing the efficacy of LECS in various gastrointestinal applications.

Onimaru et al. and Kawahara et al. reported outcomes for 48 per oral endoscopic tumor resections (POETs) [23] and 16 thoracoscopic endoscopic cooperative surgeries (TECS) for SETs in the esophagus [24]. Both POET and TECS achieved excellent R0 resection rates, with a low incidence of adverse events and no recurrence, demonstrating the effectiveness of these procedures. Therefore, POET is considered the primary option, and TECS is preferred for cases that are more extensive, malignant, or located near critical structures, such as those close to the aorta or bronchus. In addition, Onimaru et al. reported good clinical outcomes for 21 SETs in the esophagogastric junction. Though POET is the preferred approach for these junctional tumors, its technical aspects can pose challenges. The decision to use an abdominal approach, such as LECS, should take into consideration factors such as tumor size, location, malignancy characteristics, presence of delle, and the surgeon's expertise.

Oguma et al. reported eight cases of larynx‐preserving esophagectomies using a transoral flexible scope for cervical esophageal cancer. This hybrid endoscopy‐assisted, larynx‐preserving esophagectomy technique has been shown to be a valuable approach for securing an adequate proximal stump in laryngeal preservation surgery. However, there is room for improvement in achieving a more reliable resection margin and avoiding recurrent laryngeal nerve paralysis. Urabe et al. reported 19 cases of transoral robot‐assisted surgery (TORS) for superficial pharyngeal cancer. The role of the endoscopist in TORS includes diagnosing the extent of the lesion, marking the lesion, making an incision on the anal side of the lesion, and managing intra‐operative bleeding. The treatment outcomes of TORS were comparable to those of endoscopic laryngopharyngeal surgery, although a trend toward longer resection times was noted. Preoperative endoscopic diagnosis of lesion extent and peri‐anal incisions using the ESD technique of TORS may improve the accuracy of resection [25].

In conclusion, various studies have highlighted the advancements in endoscopic treatments for upper gastrointestinal diseases, achieved through collaboration between internal medicine and surgery. These findings suggest that endoscopic treatments will likely become widely adopted as standard treatment options for upper gastrointestinal diseases.

### 1.4 Endoscopic Treatment of Upper Gastrointestinal Bleeding (the 108th JGES Core Session)

A symposium on endoscopic treatment of upper gastrointestinal bleeding was held as a core session of the 108th Annual Meeting of the Japanese Gastroenterological Endoscopy Society at JDDW 2024.

Nineteen institutions in Japan submitted abstracts, of which 11 were accepted. At the beginning of the symposium, Dr. Mitsuhiro Fujishiro explained the main points of the revised endoscopic treatment guidelines for non‐variceal upper gastrointestinal bleeding in 2024 [26]. After that, Dr. Peter Draganov from the University of Florida gave an invited lecture on the current status of endoscopic hemostasis for upper gastrointestinal bleeding overseas. Re‐bleeding is one of the problems with conventional local injection and thermal coagulation methods, so OTSC and suturing devices have been introduced based on the concept of ‘oversewing’ to cover the open ulcer. Hemostatic gel and powder techniques were also introduced as primary hemostasis for extensive bleeding, such as tumor bleeding. Regarding gel and powder, they have just become available in Japan, and the indications for use should also be discussed.

A total of 11 research presentations were conducted. Five presentations were concerned with hemorrhagic duodenal ulcers, four with reducing the risk of bleeding after gastric ESD, one examined the impact of antithrombotic drug management in high‐risk bleeding endoscopic procedures, and one examined the frequency and treatment of upper gastrointestinal bleeding, including emergency cases.

Hirose et al. examined the diseases, hemostatic methods, and outcomes in upper gastrointestinal hemorrhage cases over a 16‐year period in one prefecture. Imai et al. reported on the importance of antithrombotic drug management in high‐risk bleeding procedures [27].

With regard to peptic ulcer hemorrhage, there were no studies related to gastric ulcers, and all presentations were related to duodenal ulcer hemorrhage. Kurebayashi et al. investigated whether it was possible to predict difficult endoscopic hemostasis cases in hemorrhagic duodenal ulcer using existing scoring systems, and they reported that AIMS65 [28] may be useful for predicting difficult cases of endoscopic hemostasis. Shimada et al. investigated the safety of two typical hemostatic methods, thermal soft coagulation and clipping, for hemorrhagic duodenal ulcers, and they concluded that there was no difference in adverse events or hemostatic efficacy between the two groups, and that thermal soft coagulation with its short procedure time would reduce the burden on patients. Koizumi et al. investigated the usefulness of OTSC for hemorrhagic duodenal ulcers and concluded that, although it is useful, there have been cases of rebleeding and death even with OTSC, so further consideration is needed regarding the timing of its introduction. Fukuchi et al. investigated the usefulness of absorbent local hemostatic gel for hemorrhagic duodenal ulcers and reported that it may avoid excessive thermal coagulation and could be an option for hemostatic methods in the future [29]. Fujinaga et al. reported the factors related to endoscopic hemostatic difficulty that result in conversion to interventional radiology (IVR) treatment in hemorrhagic duodenal ulcers. It appears that the management of duodenal ulcer bleeding remains an issue in clinical non‐variceal upper gastrointestinal bleeding. In the duodenum, narrow lumens, thin walls, and difficulty in endoscopic approach are thought to be factors that make hemostasis difficult. Although guidelines also recommend clipping or thermal coagulation as the first‐line hemostatic method, issues have been raised, such as the effectiveness of using new hemostatic materials, such as hemostatic gels or powder, the timing of introducing other devices, such as OTSC, and the development of an effective scoring system to assess the risk of hemostatic difficulties as an indicator of when to switch to IVR or surgery.

In terms of bleeding after gastric ESD, Iwata et al. investigated risk factors for post‐procedure bleeding and reported that prophylactic coagulation of the ulcer surface using a knife tip was not different from hemostatic forceps. Taki et al. reported that scheduled second‐look endoscopy after gastric ESD may reduce the risk of post‐procedure bleeding in patients on antithrombotic drugs. Yoshimoto et al. reported that ulcer suturing after gastric ESD in patients on antithrombotic drugs may not contribute to reducing the risk of post‐bleeding. Fujiyoshi et al. reported no superiority of potassium‐competitive acid blocker over proton pump inhibitor in the prevention of bleeding after gastric ESD in patients on antithrombotic drugs [30]. Regarding the prevention of postoperative bleeding after therapeutic endoscopic procedures such as ESD, the use of antithrombotic drugs and the location and size of the ulcer are considered important factors. After understanding the risk of postoperative bleeding, it is important to provide appropriate hemostatic prevention for the ulcer and drug therapy based on general ulcer treatment [31]. In this session, most of the studies regarding bleeding after therapeutic endoscopy examined the risk of bleeding after gastric ESD, but the number of endoscopic procedures for duodenal lesions, for which controlling bleeding is considered more difficult, is expected to increase in the future; therefore, safer and more reliable management of bleeding, including suturing of the wound, is needed.

In both peptic ulcers and postoperative ulcers, taking antithrombotic drugs is considered to be a risk factor for bleeding. In addition to the conventional hemostatic methods such as clipping and thermal coagulation, it is necessary to select the appropriate method according to the situation, including OTSC and new suturing methods, as well as gel and powder materials, whose usefulness is attracting attention.

Finally, Dr. Kazuhide Higuchi gave a special speech summarizing the reports presented at the symposium.

## 2 Conclusions

Through these core sessions, various research topics related to strictures, bleeding, and tumor treatment, including collaboration with surgery in the upper gastrointestinal tract, were discussed. We hope that the results will lead to innovative therapeutic endoscopy in the near future.

## Author Contributions

The chairpersons of each session reviewed their respective sessions, and **Mikitaka Iguchi** and **Tomoki Michida** compiled the results.

## Conflicts of Interest

The authors declare no conflicts of interest.

## Funding

The authors have nothing to report.

## Data Availability

Data sharing is not applicable to this article as no datasets were generated or analysed during the current study.
